# Multicenter Evaluation of the QIAstat-Dx Respiratory Panel for Detection of Viruses and Bacteria in Nasopharyngeal Swab Specimens

**DOI:** 10.1128/JCM.00155-20

**Published:** 2020-04-23

**Authors:** Amy L. Leber, Jan Gorm Lisby, Glen Hansen, Ryan F. Relich, Uffe Vest Schneider, Paul Granato, Stephen Young, Josep Pareja, Irene Hannet

**Affiliations:** a Nationwide Children’s Hospital, Columbus, Ohio, USA; b University of Copenhagen Hvidovre Hospital, Hvidovre, Denmark; c Hennepin County Medical Center, Minneapolis, Minnesota, USA; d Indiana University School of Medicine, Indianapolis, Indiana, USA; e Laboratory Alliance of Central New York, Liverpool, New York, USA; f TriCore Reference Laboratories, Albuquerque, New Mexico, USA; g STAT-DX Life (now a Qiagen company), Barcelona, Spain; Cepheid

**Keywords:** PCR, respiratory, multiplex

## Abstract

The QIAstat-Dx Respiratory Panel (QIAstat-Dx RP) is a multiplex *in vitro* diagnostic test for the qualitative detection of 20 pathogens directly from nasopharyngeal swab (NPS) specimens. The assay is performed using a simple sample-to-answer platform with results available in approximately 69 min. The pathogens identified are adenovirus, coronavirus 229E, coronavirus HKU1, coronavirus NL63, coronavirus OC43, human metapneumovirus A and B, influenza A, influenza A H1, influenza A H3, influenza A H1N1/2009, influenza B, parainfluenza virus 1, parainfluenza virus 2, parainfluenza virus 3, parainfluenza virus 4, rhinovirus/enterovirus, respiratory syncytial virus A and B, Bordetella pertussis, Chlamydophila pneumoniae, and Mycoplasma pneumoniae. This multicenter evaluation provides data obtained from 1,994 prospectively collected and 310 retrospectively collected (archived) NPS specimens with performance compared to that of the BioFire FilmArray Respiratory Panel, version 1.7. The overall percent agreement between QIAstat-Dx RP and the comparator testing was 99.5%. In the prospective cohort, the QIAstat-Dx RP demonstrated a positive percent agreement of 94.0% or greater for the detection of all but four analytes: coronaviruses 229E, NL63, and OC43 and rhinovirus/enterovirus. The test also demonstrated a negative percent agreement of ≥97.9% for all analytes. The QIAstat-Dx RP is a robust and accurate assay for rapid, comprehensive testing for respiratory pathogens.

## Abstract (precis)

The QIAstat-Dx Respiratory Panel (QIAstat-Dx RP) is a multiplex *in vitro* diagnostic test for the qualitative detection of 20 pathogens directly from nasopharyngeal swab (NPS) specimens. The assay is performed using a simple sample-to-answer platform with results available in approximately 69 min. The pathogens identified are adenovirus, coronavirus 229E, coronavirus HKU1, coronavirus NL63, coronavirus OC43, human metapneumovirus A and B, influenza A, influenza A H1, influenza A H3, influenza A H1N1/2009, influenza B, parainfluenza virus 1, parainfluenza virus 2, parainfluenza virus 3, parainfluenza virus 4, rhinovirus/enterovirus, respiratory syncytial virus A and B, Bordetella pertussis, Chlamydophila pneumoniae, and Mycoplasma pneumoniae.

## INTRODUCTION

Respiratory infections are common and contribute significantly to morbidity and mortality. They are also costly, being one of the leading reasons for health care visits (1, 2). Because infections with respiratory pathogens often result in symptoms that overlap between many causative agents, a definitive diagnosis requires laboratory testing. Therefore, the approach of syndromic testing has been widely adopted, with testing for multiple agents of upper respiratory infection at the same time with a single test. This panel-based approach can simplify ordering and laboratory workflow while improving sensitivity and time to result compared to older, conventional testing methods.

From a clinical perspective, the use of syndromic diagnostics can facilitate better antimicrobial stewardship by allowing antimicrobial or antiviral therapy to be given in a timely and appropriate manner (3, 4). The misuse of antibiotics in cases of viral respiratory infections is a common problem, and a rapid result for detecting a viral pathogen may prevent the unnecessary use of antibiotics. The rapid diagnosis of respiratory infections also can shorten times in the emergency room, decrease length of stay, or prevent hospitalization and allow improved patient cohorting to prevent nosocomial infections (3, 5–9).

The first multiplex respiratory panel was cleared by the FDA in 2009. This has been followed by a number of such syndromic assays. These panels vary in the number of analytes detected and the time to result, but most are designed to be simple to use and require little hands-on time (10). All current commercial multiplex assays of 5 or greater analytes include viral pathogens such as influenza A and B, respiratory syncytial virus, human metapneumovirus, adenovirus, parainfluenza virus, and rhinovirus/enterovirus. A smaller number of these panels include bacterial pathogens such as Chlamydophila pneumoniae, Mycoplasma pneumoniae, and *Bordetella* species (10).

**FIG 1:**
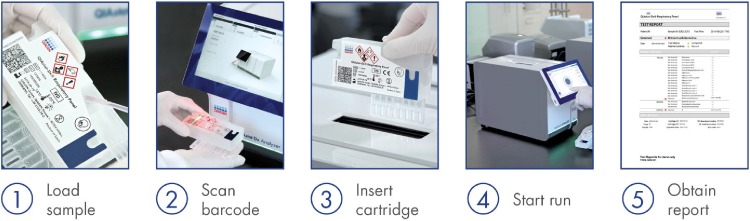
QIAstat-Dx Respiratory Panel assay workflow.

In this study, data are presented for a multicenter clinical evaluation of a new multiplex respiratory panel, the QIAstat-Dx Respiratory Panel (QIAstat-Dx RP). The QIAstat-Dx RP is a multiplexed real-time PCR test intended for use with the QIAstat-Dx system for the simultaneous qualitative detection and identification of multiple respiratory viral and bacterial nucleic acids in nasopharyngeal swabs (NPS) obtained from individuals suspected of respiratory tract infections. Each QIAstat-DX RP cartridge is run on an analyzer that consists of at least one analytical module for individual cartridge loading and one operational module with touchscreen and integrated software. Up to 4 analytical modules can be connected with one operational module (Fig. 1). The following pathogen types and subtypes are identified: adenovirus; coronaviruses 229E, HKU1, NL63, and OC43; human metapneumovirus A and B; influenza A; influenza A H1; influenza A H3; influenza A H1N1/2009; influenza B; parainfluenza viruses 1, 2, 3, and 4; rhinovirus/enterovirus; respiratory syncytial virus A and B; Bordetella pertussis; Chlamydophila pneumoniae; and Mycoplasma pneumoniae. Testing was performed on residual NPS collected in transport media. Both prospective and retrospective arms of the study are included. For all 20 analytes, performance calculations are based on comparison to an FDA-cleared/approved test.

## MATERIALS AND METHODS

### Prospective clinical specimens.

The study was conducted at six geographically distinct sites in the United States and Europe (Nationwide Children’s Hospital, Columbus, OH; Hennepin County Medical Center, Minneapolis, MN; Indiana University School of Medicine, Indianapolis IN; Laboratory Alliance of Central New York, Liverpool, NY; TriCore Reference Laboratories, Albuquerque, NM; and University of Copenhagen, Hvidovre, Denmark). Specimens were prospectively enrolled over a period of approximately 17 months (December 2017 to April 2019) and tested either fresh or after being frozen at ≤–70°C. Specimens meeting the following inclusion criterion were selected: specimen was an NPS collected in transport medium for standard-of-care (SOC) testing. The transport media used in this study were the following: Universal Transport Medium, Copan Diagnostics, Brescia, Italy, and CA, USA; MicroTest M4, M4RT, M5, M6, Thermo Fisher Scientific, MA, USA; BD Universal Viral Transport, Becton, Dickinson, NJ, USA; Universal Transport Medium, HealthLink, Inc., FL, USA; Universal Transport Medium, Diagnostic Hybrids, OH, USA; V-C-M Medium, Quest Diagnostics, NJ, USA; and UniTranz-RT Universal Transport Medium, Puritan Diagnostics, ME, USA. The specimen had to have adequate residual volume (≥2.0 ml for U.S. sites and ≥1.5 ml for Hvidovre Hospital) and been held at room temperature for less than or equal to 4 h, at 4°C for less than or equal to 3 days, or frozen at –20°C or –70°C for more than 3 days before enrollment. A waiver of informed consent requirement was obtained from the Institutional Review Boards (IRBs) at each study site for the use of residual deidentified NPS specimens.

### Retrospective (archived) clinical specimens.

Preselected frozen archive specimens were enrolled based on the identification of specific positive targets using SOC testing at each study site. Specimens were thawed and tested at each study site, in blinded fashion, with both the QIAstat-Dx RP and the comparator assay, BioFire FilmArray Respiratory Panel, version 1.7 (FARPv1.7). If the comparator assay did not confirm the preselected target as positive, the specimen was excluded from the data analysis for that target.

### Clinical and demographic data.

Data were collected for both prospective and retrospective specimens; the information included hospitalization status at the time of specimen collection, date of specimen collection, subject sex, and subject age at time of collection.

### QIAstat-Dx Respiratory Panel.

The panel includes testing for detection of adenovirus, coronavirus 229E (CoV 229E), CoV HKU1, CoVNL63, CoV OC43, human metapneumovirus A and B (hMPV), influenza A (FLU A), FLU A H1, FLU A H3, FLU A H1N1/2009, influenza B (FLU B), parainfluenza virus 1 (PIV 1), PIV 2, PIV 3, PIV 4, human rhinovirus/enterovirus (RV/EV), respiratory syncytial virus A and B (RSV), Bordetella pertussis, Chlamydophila pneumoniae, and Mycoplasma pneumoniae. Approximately 300 μl of specimen was tested according to the manufacturer’s instructions (11). The QIAstat-Dx Respiratory Panel cartridge and platform consists of automated nucleic acid extraction, reverse transcription, PCR, and fluorescence detection, with results analysis in approximately 69 min per run (i.e., per specimen); Fig. 1 shows the instrument workflow. The PCR is run for 40 cycles, and the fluorescence readings are analyzed by the result-calling algorithm (RCA) to determine positive or negative calls. The cartridge includes a full-process internal control, which is titered MS2 bacteriophage in dried form that is rehydrated upon specimen loading. This control material verifies all steps of the analysis process.

The QIAstat-Dx RP Analyzer performs automated result analysis, with each target in a valid run reported as positive or negative. The qualitative results are displayed on the instrument screen and can be printed. If the internal control fails, the software automatically will provide a result for targets that test positive, but the other panel targets will result as “invalid.” Within the software is a report to display the amplification curve for each target, for which the cycle threshold (*C_T_*) and endpoint fluorescence values are provided on the final printed report. This study was conducted with an investigational-use-only (IUO) version of the QIAstat-Dx RP that is identical to the final FDA-cleared/CE-IVD-marked version.

### Comparator testing.

Comparator testing consisted of FilmArray Respiratory Panel, version 1.7 (FARPv1.7), testing for all targets, with testing performed at the source laboratory. The assay detects adenovirus, coronavirus 229E, coronavirus HKU1, coronavirus NL63, coronavirus OC43, human metapneumovirus, influenza A, influenza A H1, influenza A H3, influenza A H1-2009, influenza B, parainfluenza virus 1, parainfluenza virus 2, parainfluenza virus 3, parainfluenza virus 4, rhinovirus/enterovirus, respiratory syncytial virus, Bordetella pertussis, Chlamydophila pneumoniae, and Mycoplasma pneumoniae.

### Results and discrepant analysis.

A QIAstat-Dx Respiratory Panel result was considered a true positive (TP) or true negative (TN) only when it agreed with the result from the comparator method (FARPv1.7). Discrepant analysis ensued when results were discordant, i.e., false-positive (FP) or false-negative (FN) results.

Discrepant analysis for all panel targets, excluding Bordetella pertussis, was performed using the NxTAG Respiratory Pathogen Panel on the Luminex MAGPIX Instrument at one clinical study site (Indiana University School of Medicine). For B. pertussis discordant analysis, the VERIGENE Respiratory Pathogens Flex Test (RP Flex) was used to detect and differentiate the following *Bordetella* species: Bordetella parapertussis*/bronchiseptica*, Bordetella holmesii, and Bordetella pertussis. This testing was performed at one clinical study site (Laboratory Alliances of Central New York).

Note that the performance data for sensitivity/positive percent agreement (PPA) and specificity/negative percent agreement (NPA) presented in this work consist of unresolved data as presented in the package insert for the FDA-cleared test; discrepancy investigation is provided but was not used to recalculate performance data.

### Workflow and time to results.

For workflow analysis, the operating procedures were compared to determine differences in the number of steps required for setup (11, 12). A total of 20 specimens were set up for analysis by both methods, and the time required from the beginning of setup to loading into the instrument was measured. In addition, a random sampling of 50 results generated on the same specimen run on the QIAstat-Dx RP and the FARPv1.7 was analyzed, and the average time to result for each platform was calculated.

### Statistical analysis.

Binomial two-sided 95% confidence intervals (CI) were calculated using the Wilson score method. Differences in median *C_T_* values were determined using Mood’s median test.

## RESULTS

### Demographics.

A total of 2,304 specimens (1,994 prospective and 310 retrospective) were included in data analysis for both arms of the study, collected from a range of geographic/demographic populations (Table 1). Overall, specimens included slightly more female than male subjects (53%, 1,222/2,304, and 47%, 1,082/2,304, respectively). The specimens were from various age groups: 33% of the specimens were from children aged 5 and under, 14% were from those aged 6 to 21, 17% were from adults aged 22 to 49, and 36% were from adults over the age of 50. For treatment setting, 32.7% (754/2,304) were obtained from hospitalized patients, 3.3% (75/2304) from those visiting the emergency department, 7.0% (161/2,304) were admitted to the intensive care unit (ICU), and 43.9% (1,012/2,304) were obtained from subjects seen in an outpatient setting. For 302 (13.1%) specimens, the location was other than those listed or unknown.

**TABLE 1:** Demographics and positivity rate for QIAstat-Dx Respiratory Panel for all prospective and retrospective samples and by age group

Parameter	Prospective sample (*n* = 1,994)		Retrospective sample^*a*^ (*n* = 310)	
	No.	% of total	No.	% of total
Demographics and location				
Male	924	46.3	158	50.8
Female	1,070	53.7	152	49.2
Outpatients	788	39.5	224	72.3
Hospitalized	686	34.4	68	21.9
Emergency	67	3.4	8	2.6
ICU	153	7.7	8	2.6
Other/unknown	300	15.0	2	0.6
Overall positivity and codetections				
Negative samples	828	41.5	11	3.5
Positive samples	1,166	58.5	299	96.5
Single detections	800	40.1	222	71.6
Codetections	366	18.4	77	24.8
Positivity by age grouping				
≤5 yr (*n* = 627)	481	24.1	137	44.2
6–21 yr (*n* = 239)	123	6.2	80	25.8
22–49 yr (*n* = 330)	174	8.7	48	15.5
50+ yr (*n* = 798)	388	19.5	34	11.5

a All retrospective samples were chosen from frozen archives based on initial standard-of-care testing and retested with the QIAstat-Dx RP and comparator.

### QIAstat-Dx RP test performance.

A total of 2,342 specimens originally were enrolled for both arms of the study (prospective, 2,018 [1,111 frozen, 907 fresh] retrospective, 324 archived frozen). A total of 24 prospective specimens were excluded for reasons related to sample stability or test performance. Fourteen retrospective samples were withdrawn because the target of interest did not confirm on repeat testing with the comparator assay (FARPv1.7).

Of the 1,994 prospective specimens tested and analyzed during the clinical evaluation, 95.9% (1,912/1,994) yielded valid results on the first attempt (i.e., first loaded cartridge). Invalid or no result was obtained for the remaining 82 specimens (4.11%). Forty-two specimens were invalid due to cartridge internal control failure (2.11%). Of these, 20 (1.00%) provided a result for positively detected targets and 22 (1.10%) had no detections. For 40 (2.00%) specimens, no results were obtained due to incomplete runs. Of these, one specimen was aborted by the user (0.05%), 21 were due to instrument errors (1.05%), and 18 were due to cartridge-related errors (0.90%).

Seventy-two of the 82 initially failed (no results or invalid) specimens yielded valid results after a single retesting using a new cartridge/specimen. The remaining 10 specimens failed on the second attempt, two due to cartridge failures, one due to instrument errors, and seven due to internal control failures. Of these internal control failures, detected pathogens were reported for four specimens. Thus, six samples (6 of 1,994 = 0.3%) did not provide valid results after a single retest, yielding a 99.7% success rate after a single retest.

### Summary of QIAstat-Dx RP findings.

**(i) Prospective specimens.** The QIAstat-Dx RP detected at least one analyte in 1,166 of 1,994 specimens tested, yielding an overall positivity rate of 58.5% (Table 1). The highest detection rate was seen in young children (≤5 years of age; 24.1%), followed by those >50 years of age (19.5%).

The summary of prospective performance characteristics for individual QIAstat-Dx RP targets is presented in Table 2. PPA and NPA were calculated with respect to the comparator method along with 95% CI. The QIAstat-Dx RP demonstrated a PPA of 91.2% or greater for all but three analytes. For FLU A H1, no PPA could be calculated. The three analytes demonstrating a PPA of <91.2% all were CoV: CoV 229E (88.9%), CoV NL63 (85.1%), and CoV 43 (89.7%). Additionally, nine analytes demonstrated a lower bound of the two-sided 95% CI of <80.0% due to few or no observations in the study. Overall, the QIAstat-Dx RP demonstrated an NPA of ≥97.9% for all analytes, with lower bounds of the two-sided 95% CI of ≥97.1%.

**TABLE 2:** Performance summary of the QIAstat-Dx Respiratory Panel for prospective specimens^*a*^

Analyte	*N*^*b*^	PPA			NPA		
		TP/(TP + FN)	%	95% CI	TN/(TN + FP)	%	95% CI
Viruses							
Adenovirus	1,986	86/90	95.6	89.1–98.3	1,880/1,896	99.2	98.6–99.5
Coronavirus 229E	1,984	8/9	88.9	56.5–98.0	1,975/1,975	100	99.8–100.0
Coronavirus HKU1	1,984	51/52	98.1	89.9–99.7	1,925/1,932	99.6	99.3–99.8
Coronavirus NL63	1,985	40/47	85.1	72.3–92.6	1,936/1,938	99.9	99.6–100.0
Coronavirus OC43	1,984	26/29	89.7	73.6–96.4	1,951/1,955	99.8	99.5–99.9
Human metapneumovirus	1,985	115/122	94.3	88.6–97.2	1,858/1,863	99.7	99.4–99.9
Rhinovirus/enterovirus	1,986	268/294	91.2	87.4–93.9	1,656/1,692	97.9	97.1–98.5
Influenza A	1,978	242/244	99.2	97.0–99.8	1,725/1,734	99.5	99.0–99.7
Influenza A H1	1,984	0/1	0.0	0.0–79.3	1,983/1,983	100.0	99.8–100.0
Influenza A H1N1\2009	1,983	80/81	98.8	98.3–99.8	1,897/1,902	99.7	99.4–99.9
Influenza A H3	1,981	156/157	99.4	93.3–99.8	1,817/1,824	99.6	99.2–99.8
Influenza B	1,983	122/129	94.6	89.2–97.3	1,853/1,854	99.9	99.7–100.0
Parainfluenza virus 1	1,984	16/17	94.1	73.0–99.0	1,964/1,967	99.8	99.6–99.9
Parainfluenza virus 2	1,984	2/2	100.0	34.2–100.0	1,982/1,982	100.0	99.8–100.0
Parainfluenza virus 3	1,987	111/113	98.2	93.8–99.5	1,869–1,874	99.7	99.4–99.9
Parainfluenza virus 4	1,984	3/3	100.0	43.8–100.0	1,979–1,981	99.9	99.6–100.0
Respiratory syncytial virus	1,985	212/220	96.4	93.0–98.1	1,760/1,765	99.7	99.3–99.9
Bacteria							
Bordetella pertussis	1,984	3/3	100.0	43.8–100.0	1,975/1,981	99.7	99.3–99.9
Chlamydophila pneumoniae	1,984	5/5	100.0	56.6–100.0	1,978/1,979	99.9	99.7–100.0
Mycoplasma pneumoniae	1,984	19/19	100.0	83.2–100.0	1,960/1,965	99.7	99.4–99.9

a These data are presented based on a comparator assay (BioFire FilmArray Respiratory Panel, version 1.7) only and do not reflect any discordant analysis. Both the fresh and frozen samples are presented together, as no statistical differences in performance were determined (data not shown).

b In instances where the internal control failed and was not resolved upon repeat, any target that was “detected” was maintained within the data set and used in performance calculations. All targets that were not detected were considered failed and excluded from the data analysis; therefore, the final *N* value will vary by analyte.

The QIAstat-Dx RP detected a total of 191 specimens, with distinctive multiple-organism detections representing 9.6% of all prospective samples. There were 166 double infections, 22 triple infections, and 3 quadruple infections. The rate of multiple detection by age group was 79.1% (151/191) for <6 years, 6.3% (12/191) for 6 to 21 years, 7.3% (14/191) for 22 to 49 years, and 7.3% (14/191) for >49 years. The three pathogens most prevalent in the multiple detections were RV/EV (108/191, 56.5%), RSV (77/191, 40.8%), and adenovirus (53/191, 27.7%).

**(ii) Retrospective specimens.** Performance characteristics for the retrospective specimens are presented in Table 3. The QIAstat-Dx RP detected at least one analyte in 299 of 310 specimens tested, yielding an overall positivity rate of 96.5% (Table 1). For the 11 negative samples, comparator testing was positive for the pathogen of interest, with retesting accruing on the same freeze-thaw cycle as the testing with QIAstat-Dx RP. With this smaller archived sample set, PPA was >90% for all but 4 targets. The lower bounds of the 95% CI for the PPA also were lower than those of the prospective group due to fewer samples being tested. Values for NPA were above 95% for all 20 targets. As these samples were preselected, prevalence was not evaluated.

**TABLE 3:** Performance summary of the QIAstat-Dx Respiratory Panel for retrospective specimens^*a*^

Analyte	No.^*b*^	PPA			NPA		
		TP/(TP + FN)	%	95% CI	TN/(TN + FP)	%	95% CI
Viruses							
Adenovirus	313	9/9	100.0	70.1–100.0	297/304	97.8	95.4–98.9
Coronavirus 229E	313	26/27	96.3	81.7–99.3	286/286	100.0	98.7–100.0
Coronavirus HKU1	313	14/14	100.0	78.5–100.0	298/299	99.7	98.1–99.9
Coronavirus NL63	312	24/24	100.0	86.2–100.0	286/288	99.3	97.5–99.8
Coronavirus OC43	310	28/28	100.0	87.9–100.0	282/282	100.0	98.6–100.0
Human metapneumovirus	313	2/2	100.0	34.2–100.0	311/311	100.0	98.7–100.0
Rhinovirus/enterovirus	313	44/49	89.8	78.2–95.5	254/264	96.2	92.3–97.9
Influenza A	313	17/17	100.0	81.5–100.0	296/296	100.0	98.7–100.0
Influenza A H1	313	0/0	NA^*c*^	NA	313/313	100.0	98.8–100.0
Influenza A H1N1/2009	312	7/8	87.5	52.9–97.8	304/304	100.0	98.9–100.0
Influenza A H3	313	8/8	100.0	67.5–100.0	305/305	100.0	98.8–100.0
Influenza B	313	1/1	100.0	20.7–100.0	312/312	100.0	98.8–100.0
Parainfluenza virus 1	307	40/40	100.0	91.2–100.0	267/267	100.0	98.8–100.0
Parainfluenza virus 2	312	3/3	100.0	100.0	309/309	100.0	98.8–100.0
Parainfluenza virus 3	313	1/4	25.0	4.6–69.9	309/309	100.0	98.8–100.0
Parainfluenza virus 4	302	22/24	91.7	74.2–97.7	278/278	100.0	98.6–100.0
Respiratory syncytial virus	313	11/12	91,7	64.6–98.5	300/301	99.7	98.4–99.9
Bacteria							
Bordetella pertussis	294	33/33	100.0	89.6–100.0	261/261	100.0	98.5–100.0
Chlamydophila pneumoniae	311	54/61	88.5	78.2–94.3	250/250	100.0	98.5–100.0
Mycoplasma pneumoniae	313	25/25	100.0	86.7–100.0	287/288	99.7	98.1–99.9

a These data are presented based on a comparator assay (BioFire FilmArray Respiratory Panel version 1.7) only and do not reflect any discordant analysis.

b In instances where the internal control failed and was not resolved upon repeat, any target that was “detected” was maintained within the data set and used in performance calculations. All targets that were not detected were considered failed and excluded from the data analysis; therefore, the final *N* value will vary by analyte.

c NA, not applicable.

**(iii) Comparator analysis and discrepancy investigation.** There was a total of 45,895 analyzable QIAstat-Dx RP pathogen results for the 2,304 specimens (prospective and retrospective). The overall percent agreement between QIAstat-Dx RP and the comparator testing was 99.5% (45,662/45,895). There were 2,075 detected pathogen results with the QIAstat-Dx RP; the comparator method was positive for 2,026 pathogen detections. The overall PPA with respect to the comparator method was 95.5% (1,934/2,026). Data for the median *C_T_* values for all positive detections for the QIAstat-Dx RP are presented in the supplemental material (Table S1).

There were 43,871 “not detected” results with the QIAstat-Dx RP; the comparator method was negative for 43,920 analytes. The overall NPA with respect to the comparator method was 99.7% (43,728/43,869).

For the viral analytes, QIAstat-Dx RP detected a total of 1,923 viral analytes compared to 1,880 for FARPv1.7. Using the comparator as the true result, the overall PPA and NPA were 95.5% (1,795/1,880) and 99.7% (32,101/32,117), respectively, for virus targets. Using the comparator as the true result, the overall PPA and NPA were 92.3% (36/39) and 99.9% (6402/6409), respectively, for all bacterial targets.

Using comparator testing as the true result, there were 141 FP detections and 92 FN detections overall; additional discrepancy analysis was performed for 214 (91.8%) of these false detections. For 62 of the 141 FP cases (44%), along with 30 of the 92 FN cases (33%), there was supportive evidence for the QIAstat-Dx RP result, bringing the adjudicated overall concordance for the positive and negative results to 98.5% and 99.7%, respectively. A summary of the discrepancy investigation is presented in Table 4.

**TABLE 4:** Results of discrepant investigation for QIAstat-Dx Respiratory Panel, prospective and retrospective specimens^*n*^

Result disposition based on initial testing vs comparator	False negatives^*a*^			False positives		
	QDRP result, total FN	Discrepant investigation outcome		QDRP result, total FP	Discrepant investigation outcome	
		QDRP confirmed^*b*^ (TN)	QDRP unconfirmed (FN)		QDRP confirmed^*b*^ (TP)	QDRP unconfirmed (FP)
Viruses						
Adenovirus^*c*^	4	1	3	23	9	14
Coronavirus 229E^*d*^	2	0	2	0		
Coronavirus HKU1	1	1	0	8	0	8
Coronavirus NL63^*e*^	7	0	7	4	1	3
Coronavirus OC43^*f*^	3	3	0	4	3	1
Human metapneumovirus	7	3	4	5	3	2
Rhinovirus/enterovirus^*g*^	31	9	22	46	18	28
Influenza A^*h*^	2	1	1	9	3	6
Influenza A H1^*i*^	1	0	1	0		
Influenza A H1/2009	2	0	2	5	3	2
Influenza A H3	1	0	1	7	7	0
Influenza B^*j*^	7	0	7	1	1	0
Parainfluenza virus 1	1	1	0	3	3	0
Parainfluenza virus 2	0			0		
Parainfluenza virus 3	5	2	3	5	3	2
Parainfluenza virus 4	2	1	1	2	2	0
Respiratory syncytial virus	9	7	2	6	3	3
Bacteria						
Bordetella pertussis^*k*^	0			6	1	5
Chlamydophila pneumoniae^*l*^	7	1	6	1	1	0
Mycoplasma pneumoniae^*m*^	0			6	1	5
						
Total	92	30	62	141	62	79

a Result disposition based on initial testing with QDRP versus comparator testing with BioFire FilmArray Respiratory Panel, version 1.7.

b QIAstat-Dx RP confirmed, the results of discrepant analysis supported the original QIAstat-Dx Respiratory Panel result as true negative or true positive. QIAstat-Dx RP unconfirmed, the results of discrepant analysis did not support the original QIAstat-Dx Respiratory Panel result, and the result was considered false negative or false positive.

c Two FP adenovirus specimen did not undergo discordant analysis and were considered unconfirmed FP.

d Two FN coronavirus E229 specimen did not undergo discordant analysis and were considered unconfirmed FN.

e Two FP coronavirus NL63 specimen did not undergo discordant analysis and were considered unconfirmed FP.

f One FN coronavirus OC43 specimen did not undergo discordant analysis and was considered unconfirmed FN.

g Three FN rhinovirus/enterovirus specimen did not undergo discordant analysis and were considered unconfirmed FN.

h Three FP influenza A samples were not available for discrepancy testing and were considered unconfirmed FP.

i Non-2009 H1 has not been in circulation since being replaced by the 2009 H1; thus, the discrepancy test result for the FN 2009-H1 sample is likely false.

j One FN influenza B sample was not available for discrepancy testing and was considered unconfirmed FN.

k One FP Bordetella pertussis sample was not available for discrepancy testing and was considered unconfirmed FP.

l Two FN Chlamydophila pneumoniae samples were not available for discrepancy testing and were considered unconfirmed FN.

m One FP Mycoplasma pneumoniae sample was not available for discrepancy testing, and another FP sample did not produce a valid result with the discrepancy method; both FP results were considered unconfirmed FP.

n QDRP, QIAstat-Dx Respiratory Panel; TN, true negative; FN, false negative; TP, true positive; FP, false positive.

**(iv) Workflow and time to results.** A review of the procedure showed that the steps for setting up the pouches to loading in the instrument differed between the two platforms, with the specimen being pipetted directly into the QIAstat-Dx, while the FA RPv1.7 required the addition of both sample and a diluent using injection vials for reagent hydration and sample preparation in addition to a transfer pipette for manipulating the specimen. Timed studies for the setup of 20 pouches by two operators, from specimen to loading, took an average of 35 s for the QIAstat-Dx RP and 115 s for the FARPv1.7.

The average time to results for the 50 paired runs as determined by each instrument was 69.1 ± 0.8 min for QIAstat-Dx RP and 63.4 ± 0.5 min for FARPv1.7.

## DISCUSSION

This evaluation of the QIAstat-Dx RP demonstrated the performance of the test in a large multicenter study using 2,304 residual NPS specimens with 45,895 results generated. This new respiratory multiplex panel can be used to aid in the diagnostic testing of respiratory infections. In this trial, the number of prospective positive detections was relatively high for most pathogens, with the exception of CoV 229E, PIV 4, and B. pertussis (all with *N* < 5). No detections were found for C. pneumoniae, PIV 1, and FLU A H1, which was not circulating during the study period. The QIAstat-Dx RP testing system was shown to be reliable, with few failures (95.3% success on the initial test attempt for the prospective samples tested), and rapid, with results available in approximately 69 min. The data presented here, along with the testing of contrived specimens, were used as part of the regulatory submissions for the QIAstat-Dx RP, which received *de novo* 510(k) clearance in the United States in May 2019 (11).

Taken in total, the QIAstat-Dx RP performance was comparable to that of the FARPv1.7, with an overall percent agreement of 99.5%. In the prospective cohort, the QIAstat-Dx RP demonstrated a PPA of 94.0% or greater for detection of all but four analytes: CoV 229E, CoV NL63, and CoV OC43, and RV/EV. The test also demonstrated an NPA of ≥99.6% for all analytes. The discordant analysis showed that both assays appear to generate “false” results, as would be expected. The NxTAG assay, used for discordant analysis, is very similar to both of these assays, being a multiplex respiratory panel. Thus, for the discordant analysis, a true result was determined by the best of two out of three test results.

Viruses are a common cause of respiratory infections in both adult and pediatric populations, which was also seen in our study cohort. While the QIAstat-Dx RP had a higher number of positive viral detections overall than FARPv1.7 (1,645 versus 1,610), for individual targets, there was increased sensitivity found with both assays depending on the analyte. Viral detections were notably higher than those of the bacterial targets among the prospective specimens (1,645 viral versus 39 bacterial detections).

### Rhinovirus/enterovirus.

The most common viral analyte was RV/EV, with a total of 304 positive detections. The extensive diversity within the rhinoviruses means that most molecular assays, including QIAstat-Dx RP and the comparator assay, target the 5′ untranslated region (UTR). This region is highly conserved among all rhinoviruses and enteroviruses, causing cross-reactivity between assays for the two viruses and making their differentiation difficult (13). RV/EV was also the target showing the highest number of discordant results. The discordant specimens were analyzed with the NxTAG Respiratory Pathogen Panel, another FDA-cleared multiplex that targets the 5′UTR. Therefore, no definitive resolution of the type of virus (rhinovirus versus enterovirus) was made for the FP and FN samples.

### Adenovirus.

For adenoviruses, QIAstat-Dx RP is designed to detect genogroups B, C, and E, the types most commonly associated with respiratory infections. It will also detect, to some degree, genotypes A, D, F, and G, as evidenced by contrived testing with typed strains (11). The FARPv1.7 was also designed for the detection of genotypes B, C, and E. Both tests use the hexon gene as the target. The differences in performance between these two tests in the present study may be related to specific primer and probe sequence differences or the level of sensitivity and specificity of the assays for the many different serotypes of adenovirus (prior studies have demonstrated that a significant number of adenoviruses from upper-respiratory-tract samples may be in genogroups A, D, and F and could be missed by tests that are not designed for broad coverage of adenoviruses) (14, 15). Some recent data suggest that broadened inclusivity targeting the nonrespiratory types improves clinical assay performance (16).

### Coronavirus.

The QIAstat-Dx RP assay has 4 distinct targets for detection of CoV. Three of four of these targets (Table 2) had PPA of <90%, which were the lowest values for all analytes in the prospective analysis. In contrast, the retrospective CoV specimens showed better positive agreement with all targets of >96% (Table 3). It is unclear why there were differences in performance in the two arms of the study. There was a relatively small number of positive detections in general. In addition, the FP sample did have significantly higher *C_T_* values than the TP for all samples (see Table S1 in the supplemental material), suggesting that the FP were related to the low level of virus. The level of virus in these FN specimens cannot be estimated, as no semiquantitative value, such as *C_T_*, is provided with the FARPv1.7.

### Influenza viruses.

Among the viruses detected in this multiplex panel, there is substantial evidence that the rapid molecular diagnosis of influenza virus infections impacts patient outcomes for both adult and pediatric populations (3, 6, 9). The QIAstat-Dx RP has a total of 4 targets for the detection of FLU A: a paninfluenza A target and specific targets for 3 subtypes, influenza A H1, influenza A H1 2009, and influenza A H3. Of the 251 influenza A-positive detections, a total of 248 (98.8%) had additional subtype-specific detections (85 specific detections for H1N1 2009 and 163 for H3). There were no detections of seasonal H1N1. Three (1.2%) influenza A positives had no associated specific detections. This could be due to virus levels below the limit of detection for the type-specific assays. However, it indicates the detection of a novel influenza A type, and this should be considered when seen in clinical use (17, 18). For FLU B virus, there is a single target designed to detect the two sublineages of the virus (B/Victoria/2/87-like [Victoria lineage] and B/Yamagata/16/88-like [Yamagata lineage]). There were 7 FN results with the QIAstat-Dx RP and 1 with FARPv1.7, which may reflect differences in sensitivity related to the viral strains. Because the comparator does not provide any semiquantitative value, it is difficult to determine the relative level of virus in the 7 QIAstat-Dx FN; however, the 1 FN for the FARPv1.7 had a *C_T_* value of 20.4, suggesting it did have a significant amount of virus present.

A relatively small number of bacterial detections were found in the prospective cohort, as has been seen in other studies with multiplex testing (16, 19). The most common of the bacterial targets was M. pneumoniae, with 24 detections, more than the 19 detected with FARPv1.7. However, it should be noted that the use of an NP specimen for the detection of M. pneumoniae may be suboptimal, particularly when diagnosing lower-respiratory-tract infection (20, 21). For B. pertussis, discrepancies between the QIAstat-Dx RP and the comparator method were not unexpected, as the QIAstat-Dx RP targets the multicopy insertion sequence (IS*481*) that is present in several *Bordetella* species (B. pertussis, *B. holmesii*, and B. bronchiseptica), whereas the comparator targets the single-copy promoter region of the pertussis toxin gene and is designed to be specific to the detection of B. pertussis. The use of the single-copy toxin gene target has been shown to be less sensitive than the use of IS*481* (22, 23). The assay used for discordant analysis calls out the individual *Bordetella* species (B. pertussis, *B. parapertussis*, and *B. holmesii*). The B. pertussis target is also the toxin promoter region and, thus, would be a single-copy gene. A total of 6 QIAstat-Dx RP FP results were found for B. pertussis for both arms of the study (Table 4). Five of these samples were available for further analysis; only one was confirmed using the discordant testing with a *C_T_* value of 31.6. In examining the *C_T_* values for all the detections in the clinical trial, the discordant detections had a significantly higher median *C_T_* value than the concordant positive detections (TP median *C_T_*, 26.2; FP median *C_T_*, 33.0; *P* = 0.008) (Table S1). Thus, some of the FP may have been missed by both the comparator and discordant analysis assays based on the lower sensitivity of a single-copy gene target.

As with other multiplex respiratory panels, the QIAstat-Dx RP allows for the detection of multiple pathogens representing coinfections. The rate of codetections was highest in the pediatric patients <6 years of age, and the most common analytes in the codetections were RV/EV, RSV, and adenovirus. Similar findings have been reported for other multiplex respiratory panels (16, 19). More data are needed on the impact of codetections on outcomes; however, it may be useful for infection control purposes to allow better cohorting or isolation of infected patients (24).

The QIAstat-Dx RP workflow is very simple and the footprint of the instruments is small, measuring 20.3 cm (width) by 32.6 cm (height) by 51.7 cm (depth) for one operational module plus one analytical module (8.0 in [width] by 12.8 in [height] by 20.4 in [depth]) (Fig. 1). Compared to FARPv1.7, QIAstat-Dx RP involves only one step to pipette the specimen into the cartridge for loading, as there is no buffer added or any other manipulations required. This lessens manipulation and may help to reduce contamination. The run times are similar, lasting, on average, 69 and 63 min for the QIAstat-Dx RP and FARPv1.7, respectively. Both platforms allow for testing of one pouch at a time per module. In terms of reliability, the initial rate of invalid or no results on first testing for the prospective samples was 4.1% and after a second test was 0.7%. This invalid rate is comparable to those of other multiplex platforms currently available (16). A significant benefit of the system is that it allows the user to obtain a *C_T_* value for each detected pathogen and the internal control. The comparator assay does not allow the user to see *C_T_* values. These values, while not truly quantitative, do allow a semiquantitative assessment of target amounts that can be useful in troubleshooting or other quality control measures.

There are some limitations to this study. For the prospective arm, some specimens were tested fresh but some were frozen at ≤–70°C prior to testing. However, data indicated that the frozen storage did not significantly affect performance (11). The study period bridges 17 months and includes two respiratory seasons; however, variations in circulating strains, particularly influenza A viruses, may be limited. Because the prevalence of some analytes was low in the prospective cohort, frozen retrospective samples were used to increase the numbers for positive detections. As stated above, freeze-thawing did not appear to affect performance in terms of prevalence. However, for the retrospective samples, all were tested with both the test and comparator assays on the same freeze-thaw cycle to remove this as a confounder. Overall, the percentage of discrepant results versus the comparator methods was low.

In summary, the QIAstat-Dx RP demonstrated good comparative performance in this large multicenter clinical trial and represents a new alternative for multiplex respiratory testing. It is a robust and accurate assay for rapid and comprehensive testing for respiratory pathogens from nasopharyngeal swab specimens.

## Supplementary Material

Supplemental file 1
