# Supplementary material for: Multicenter Evaluation of the QIAstat-Dx Respiratory Panel for Detection of Viruses and Bacteria in Nasopharyngeal Swab Specimens
Source: J Clin Microbiol. 2020 Apr 23;58(5):e00155-20. doi: 10.1128/JCM.00155-20 (PMC7180242; doi:10.1128/JCM.00155-20)
Supplement: Supplemental file 1 [file JCM.00155-20-s0001.pdf]

Supplemental Data:

TABLE S1 Median Cycle Threshold (Ct) values for the True Positive (TP) and False Positive (FP) Detections with the QIAstat-Dx Respiratory Panel – Data compiled from results before discrepant analysis.

Analyte	TP[a]	FP	
	Median Ct (IQR, n)[b]	Median Ct (IQR, n)	*P*value[c]
Adenovirus	29.0 (22.2-32.8, n=92)	34.1 (33.4-35.6, n= 23)	**0.001**
Coronavirus 229E	28.9 (25.0-30.8, n=34)	-	NA
Coronavirus HKU1	27.5 (22.0-30.6, n=65)	34.0 (32.0-36.0, n=8)	**0.002**
Coronavirus NL63	29.7 (24.6-32.2, n=64)	34.9 (33.2-35.4, n=4)	**0.039**
Coronavirus OC43	25.1 (20.9-29.7, n=54)	34.1 (31.6-34.3, n=4)	**0.038**
Human Metapneumovirus	25.9 (20.8-29.8,n=116)	34.6 (32.6-35.1, n=5)	**0.017**
Rhinovirus/Enterovirus	28.4 (24.5-30.7, n=312)	32.4 (30.6-34.2,n=46)	**<0.0001**
Influenza A	27.3 (22.9-30.9, n=259)	34.7 (34.2-36.4, n=9)	**0.016**
Influenza A H1	-	-	NA
Influenza A H1N1\2009	27.1 (23.7-30.0, n=88)	30.3 (27.7-35.2, n=5)	0.160
Influenza A H3	25.7 (20.5-29.6, n=164)	33.5 (29.5-29.6, n=5)	**0.022**
Influenza B	24.5 (21.4-28.2, n=123)	(20.7)[c]	NA
Parainfluenza Virus 1	27.7 (24.1-31.6, n=57)	35.7 (34.0-36.4, n=3)	**0.006**
Parainfluenza Virus 2	30.0 (27.3-34.0, n=5)	-	NA
Parainfluenza Virus 3	21.05 (18.2-27.5, n=112)	34.7 (32.1-35.5, n=5)	**0.021**
Parainfluenza Virus 4	27.1 (25.4-31.4, n=25)	30.0 (NA. n=2)	0.127
Respiratory Syncytial Virus	23.0 (18.5-28.8, n=224)	34.2 (30.3-35.1, n=5)	**0.021**
Bordetella pertussis	26.2 (22.9-28.8, n= 36)	33.0 (30.9-34.2, n=6)	**0.008**
Chlamydiophila pneumoniae	30.9 (27.9-32.8, n=56)	(35.6)[c]	NA
Mycoplasma pneumoniae	25.4 (23.6-28.4, n=44)	34.1(33.2-35.7, n=6)	**0.009**

[a]True positive and False positive based on comparator assay (FA RPv1.7).
[b]*P*values for differences between median values determined using Mood's Median Test. Significant values are in bold.
[c] Single value for FP
IQR, Interquartile range; NA, not applicable
